# Supplementary material for: Development and Evaluation of an Index to Measure the Ability to Get Vaccinated for COVID-19
Source: Vaccines (Basel). 2023 Feb 3;11(2):342. doi: 10.3390/vaccines11020342 (PMC9966146; doi:10.3390/vaccines11020342)
Supplement: Supplementary file 1 [file vaccines-11-00342-s001.zip › vaccines-2179203-supplementary.pdf]

Supplemental Materials: Internal Consistency and Factor Analysis

Although we prefer to conceptualize ability as an induced rather than latent variable as discussed in the main manuscript, we nevertheless carry out some conventional internal consistency analyses. First, we generated and examine the matrix of pairwise correlations among the twelve ability indicators. Second, we conducted an exploratory factor analysis of these items using the principal factors method, examining the eigenvalues to gain insight into the dimensionality of the items, and presenting factors loadings from the one- and two-factor solutions. We use an oblique promax rotation for the two-factor solution, as it seemed arbitrary and even implausible to assume that the factors would be uncorrelated with one another. Third, we computed Cronbach's alpha for the full twelve-item scale and the two subscales implied by the exploratory factor analysis. We present the results of those analyses here for the benefit of readers who prefer to conceptualize ability as a latent variable.

Supplementary Table S1 displays the pairwise correlations among the twelve ability index items. Most pairs of items are moderately, positively, and statistically significantly associated with one another. Supplementary Table S2 shows the factor analysis and internal consistency results. The eigenvalues displayed in the second column suggest either a one-dimensional or two-dimensional factor structure, depending upon which of the commonly used criteria one uses. Columns 3 shows the factor loadings from the one factor solution, and columns 4 and 5 show the loadings from the oblique promax rotated two-factor solution. For the one-factor solution, the loadings range from 0.29 for item 1 to 0.63 for item 6. The two-factor solution achieves very close to simple structure, with items 1, 4, 7, 8, 9, 10, and 12 loading on the first factor, and items 2, 3, 5, 6, and 11 loading on the second factor. There is no clear thematic consistency in the content of items loading on one factor versus the other, but notably all of the items that load on the first factor involve barriers or obstacles to vaccination, whereas all of those that load on the second factor involve the absence of such barriers or the presence of supports for vaccination. The bottom row of Supplementary Table S2 shows that the full twelve-item index and the seven- and five-item sub-indexes based on the two-factor model have Cronbach's alpha values above the conventional 0.70 threshold for acceptability.

Supplementary Table S1. Pairwise Pearson Correlations among the Ability Index Items (n=781)

	1.	2.	3.	4.	5.	6.	7.	8.	9.	10.	11.	12.
1.	1.00											
2.	0.00	1.00										
3.	0.03	0.33***	1.00									
4.	0.23***	0.15***	0.21***	1.00								
5.	0.04	0.38***	0.41***	0.14**	1.00							
6.	0.09*	0.34***	0.46***	0.21***	0.42***	1.00						
7.	0.17***	0.04	0.19***	0.39***	0.07	0.32***	1.00					
8.	0.25***	0.09*	0.15***	0.27***	0.06	0.22***	0.25***	1.00				
9.	0.19***	0.12**	0.21***	0.26***	0.10**	0.23***	0.24***	0.42***	1.00			
10.	0.23***	0.12**	0.16***	0.26***	0.13**	0.10**	0.20***	0.25***	0.33***	1.00		
11.	0.10**	0.24***	0.36***	0.15***	0.34***	0.45***	0.20***	0.14***	0.17***	0.10***	1.00	
12.	0.17***	0.05	0.12**	0.33***	0.00	0.13***	0.28***	0.27***	0.30***	0.21***	0.08*	1.00

Note: *p<0.05, **p<0.01, ***p<0.001.

Supplementary Table S2. Factor Analysis and Internal Consistency Findings (n=781)

		One Factor Model	Two Factor Model	
	Eigenvalues	Loading	Loading 1	Loading 2
1. My family and household responsibilities make it difficult for me to find time to get a COVID-19 vaccine.	2.61	0.29	0.41	-0.07
2. I can leave my work long enough to get the COVID-19 vaccine.	1.07	0.39	-0.05	0.52
3. The COVID-19 vaccine is affordable to me.	0.22	0.55	0.07	0.60
4. I would lose income by taking time to get the COVID-19 vaccine.	0.12	0.50	0.53	0.08
5. My supervisor would support my getting the COVID-19 vaccine.	0.05	0.45	-0.10	0.65
6. The COVID-19 vaccine is conveniently available to me.	-0.05	0.63	0.11	0.65
7. I have to travel a long way to get the COVID-19 vaccine.	-0.09	0.47	0.48	0.09
8. I am worried that side effects of the COVID-19 vaccine would make it difficult for me to fulfill my work or family responsibilities.	-0.13	0.46	0.56	0.00
9. The decision to get the COVID-19 vaccine is difficult.	-0.15	0.50	0.55	0.05
10. I have a spouse/partner who would not approve of me getting a COVID-19 vaccination.	-0.16	0.39	0.45	0.02
11. I know where to go to get the COVID-19 vaccine.	-0.20	0.39	0.06	0.53
12. It would require a big change in my daily routine to get the COVID-19 vaccine.	-0.23	0.38	0.53	-0.07
		$\alpha = 0.76$	$\alpha = 0.71$	$\alpha = 0.75$
